# Supplementary material for: Role of inflammation in depression and anxiety: Tests for disorder specificity, linearity and potential causality of association in the UK Biobank
Source: eClinicalMedicine. 2021 Jun 26;38:100992. doi: 10.1016/j.eclinm.2021.100992 (PMC8413248; doi:10.1016/j.eclinm.2021.100992)
Supplement: Supplementary file 2 [file mmc2.docx]

# Caption for supplementary material

1. Supplementary appendix
2. STROBE checklist
